# *Lettowia*, a new genus of Vernonieae from East Africa (Asteraceae)

**DOI:** 10.3897/phytokeys.25.5556

**Published:** 2013-07-19

**Authors:** Harold Robinson, John J. Skvarla

**Affiliations:** 1 Dept. of Botany, MRC 166, National Museum of Natural History, P.O. Box 37012, Smithsonian Institution, Washington, D.C. 20013-7012; 2 Dept. of Botany and Microbiology, Oklahoma Biological Survey, University of Oklahoma, Norman, Oklahoma, 73018-6131, USA

**Keywords:** East Africa, *Lettowia*, new genus, triporate pollen

## Abstract

A new genus, *Lettowia* H. Rob. & Skvarla is named for the single East African species originally described as *Vernonia nyassae* Oliv. Its pollen is lophate and triporate, with a perforated tectum restricted to the muri. The new genus is placed near *Vernoniastrum* in the subtribe Erlangeinae.

## Introduction

Since *Vernonia* Schreb. is a strictly American and mostly eastern North American genus (Robinson 1999a, b), a continuing effort is under way to properly reassign the species of the Eastern Hemisphere that have been erroneously placed in that genus. In previous efforts to resolve the species of Vernonieae from Africa, one species, *Vernonia nyassae* Oliv., was examined but left untreated. In a careful analysis of East African Vernonieae, Jeffrey (1988) keyed *Vernonia nyassae* among species that are now mostly placed in the subtribe Erlangeinae, and the species was listed among those now mostly placed in the genus *Vernoniastrum* H. Rob. Robinson (1999a) studied but did not treat the species; however, its possible relation to *Vernoniastrum* had been considered. The failure to include the species in that genus was because of the lack of the distinctive bands of idioblasts in the achene walls, and the presence of an inflorescence of single elongate unbranched erect scapes each bearing a single capitulum. *Vernoniastrum* has branched inflorescenses with cauline leaves, and has bands of idioblasts in the achene wall that are visible with a hand-lens. For these reasons, the species is considered here to be related to *Vernoniastrum* but placed outside of that, hence in need of recognition as a new genus.

The present review includes a full description of the plant, including SEM studies of pollen, and a formal description of the new genus.

## Preparation of pollen

Pollen from dried buds was obtained from herbarium sheets at the U.S. National Herbarium in Washington, D.C. Detailed light microscope analyses under a Wild light microscope using oil and immersion optics were made of pollen in the dry condition as well as after immersion in Hoyer’s solution (Anderson 1954). Scanning electron microscope (SEM) observations were made after acetolysis treatment (Erdtman 1960). *Lettowia nyassae* pollen was examined with a Hitachi S-570 SEM (at the United States National Museum of Natural History) after coating with gold/palladium. *Vernoniastrum nestor* pollen was examined with a Zeiss Neon 40 EsB dual beam SEM/FIB after metal coating treatments with osmium thiocarbohydrazide and gold/palladium (Chissoe et al. 1995, 1996). Images were digitally processed and the final plates prepared using Adobe Photoshop 7.

## Taxonomic treatments

### Lettowia

H.Rob. & Skvarla gen. nov.

urn:lsid:ipni.org:names:77130232-1

http://species-id.net/wiki/Lettowia

Figs 1 , 2A, B

#### Type.

*Vernonia nyassae* Oliv.

#### Description.

Erect or decumbent perennial herbs from creeping rhizome or perennial root crown, all becoming erect, rosettiform, with erect pedunculate scapiform inflorescence; hirsute or pilose with long white hairs, hairs of stems, leaves and peduncles with cells uniseriate and with few short basal cells and long, acicular, rather stiff apical cell. Leaves alternate in loose basal rosette, mostly 2–4 cm long, petiole narrow, blade obovate, to 9 cm long and 1.5 cm wide, apically obtuse, cuneate into petiole at base, margins entire, slightly paler abaxially, pilose on margins and both surfaces, more densely pilose abaxially, few glandular dots adaxially, numerous dots abaxially. Scape mostly 5–11 cm long, densely and stiffly hirsute, sometimes with small bract near middle, bearing 1 terminal head. Heads broadly campanulate, up to 2 cm high, 1.5–1.8 cm broad. Involucre with ca. 15–20 persistent, mostly subequal ovate-lanceolate bracts in ca. 2 series, up to ca. 1.5 cm long, apices acute, without acumination, densely pilose outside with long simple hairs, with 3 longitudinal veins, margins narrowly scarious, sometimes reddish. Receptacle epaleaceous; florets ca. 40 in a head; corollas lavender, ca. 12 mm long, narrowly funnelform distally from slender basal tube, tube ca. 7 mm long, with stalked narrowly capitate glands outside, throat ca. 0.7 mm long, lobes linear, ca. 4.3 mm long, with glandular dots outside and numerous stiff uniseriate hairs distally; anther thecae ca. 3 mm long, with tapering bases, with short clavate tails, apical appendage oblong, glabrous, with tenuous cell walls; style base with narrow annular node; with acicular sweeping hairs restricted almost completely to branches; achenes weakly 5-costate, densely sericeous on and between ribs with long setulae, setulae slightly split at tips, glandular dots present near base, without evident idioblasts, raphids linear; carpopodium narrowly annuliform, with small quadrate cells; pappus of ca. 40 persistent barbellate bristles ca. 8 mm long, mostly of even width, tapering at extreme tips, with outer series of short narrowly lanceolate squamae. Chromosome number not known. Chemistry not known.

Pollen grains of *Lettowia nyassae* (Fig. 2A, B) ca. 55 µm in diam in fluid, ca. 45 µm dry, echinolophate, triporate, muri shortly echinate with 2–3 spicules on each mural segment, perforated tectum restricted to the muri. The lacunae are irregular in position and rather irregular in shape. The baculae are elongate, in a mostly single partially unaligned series under each murus, and they are firmly attached to the footlayer. In these features, the pollen is nearly like that of *Vernoniastrum* (Fig. 2A, B), and the relationship to that genus in the subtribe Erlangeinae is assumed. In direct comparison with the single species of *Vernoniastrum* for which SEM study of the pollen is available (Fig. 2C–F), a number of subtle differences can be seen. In *Lettowia*, the lacunae are larger in general and less numerous (ca. 25–30 in *Lettowia* versus 35–40 in *Vernoniastrum*), the spicules on the muri are shorter and less numerous, and the baculae under the muri are mostly in a single row. In both genera there are lacunae that are elongate as if two lacunae are joined, but these do not seem to be aligned in positions where colpi might be located.

**Figure 1.**
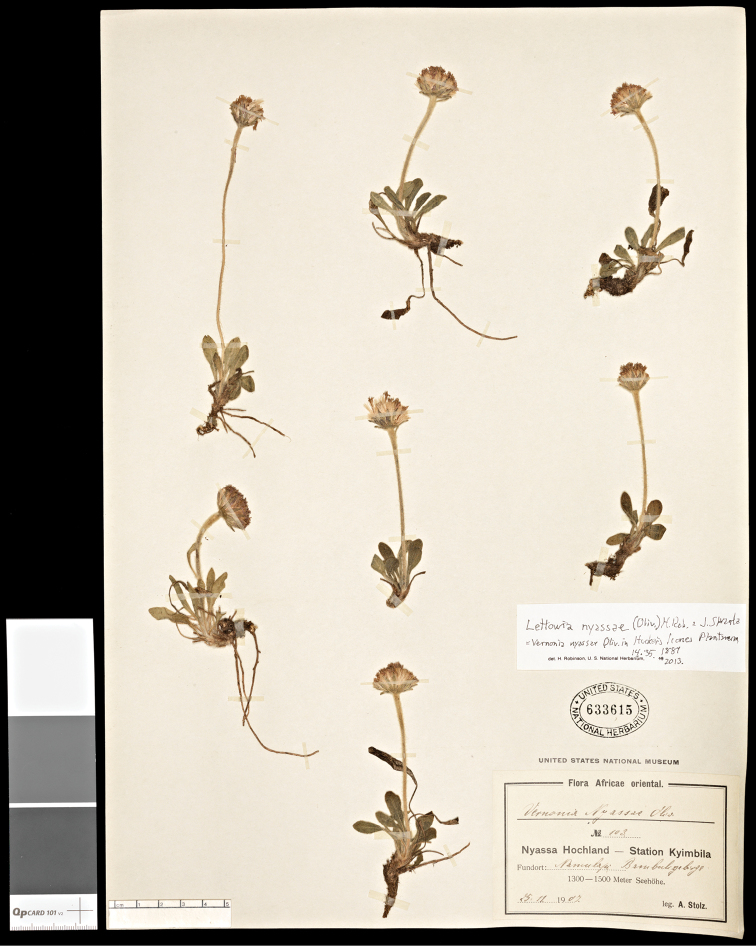
Herbarium specimen of *Lettowia nyassae* (Oliv.) H. Rob. & Skvarla (*A. Stolz 103*, US).

**Figure 2.**
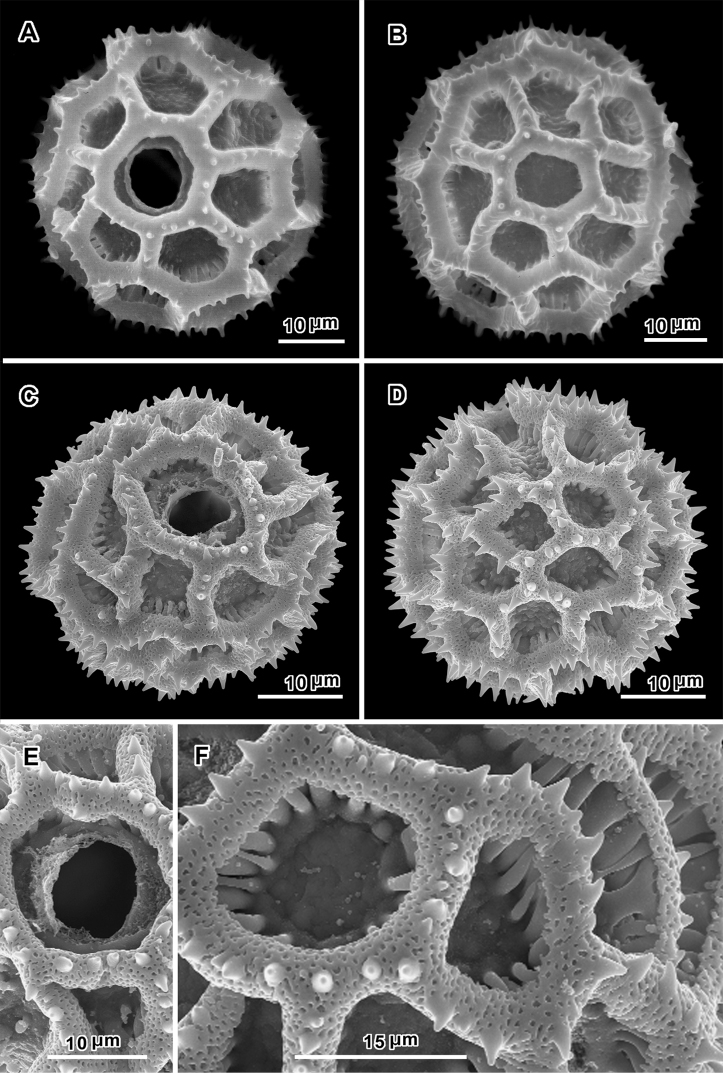
**A–F** SEM of pollen. **A–B** *Lettowia nyassae* (Oliv.) H. Rob. & Skvarla (*A. Stolz 103*, US) **A** View with a pore **B** Surface lacking pores **C–F** *Vernoniastrum nestor* (S. Moore) H. Rob. (Burundi, Prov. Burundi, Gihofi (Mosso), 20 May 1980, *Reekmans 9185*, US) **C** View with a pore **D** Surface lacking pores **E** Closer view of lacuna with a pore **F** Closer view of surface without pores showing extra rows of baculae.

#### Etymology.

The genus is named for Colonel Paul von Lettow-Vorbeck, 1870–1964 (Gunther 1956; Hoyt 1981; Lettow-Vorbeck 1920), who led the defense of German East Africa in WWI until the surrender of Germany, and was remembered fondly by the people of the former German colony on a return visit after WWII.

The genus contains the following single species.

### Lettowia nyassae

(Oliv.) H. Rob. comb. nov.

urn:lsid:ipni.org:names:77130233-1

http://species-id.net/wiki/Lettowia_nyassae

Figs 1 , 2A, B

Vernonia nyassae Oliver in J.D. Hooker, Icon. Pl. 14(2): 36, t. 1349B. 1881. Type: Tanzania, Higher plateau north of Lake Nyassa, *Thomson s.n.* (K, photo in Smith 1971: 65–67, fig. 45A–E).

#### Distribution.

The species occurs primarily in the area of southwestern Tanzania north of lake Nyassa, and also in eastern Zambia, Ndola, Oct 1906, *C.F.E. Allen 363 * (SRGH). Smith (1971) cited an outlying population of *Vernonia nyassae* in Transvaal (*Codd & Winter 3291*, K). Examination of the voucher specimen at Kew shows that it belongs to *Vernonia thodei* Phillips (now in *Pseudopegolettia* H. Rob., Skvarla & Funk, in prep.), a totally distinct entity with a different non-lophate form of pollen.

Jeffrey (1988) cited a specimen of *Vernonia nyassae* from the region T4, *Bally 7496*, from Ugalla R. (K), which is “smaller than specimens from T7 in all floral parts; it may be simply depauperate, or may indicate a populational difference.”

Smith (1971) cited the following specimens as seen from within the range of the species: **Tanzania:** Sao Hill, 6200 feet, Feb 1959, *A.M. Watermeyer 32* (K). Mbeya Distr., Slopes of Mbeya Mt., 9000 feet, 25 Sep 1936, *B.D. Burtt 6331* (K). Southern Highlands Prov., Njombe Distr., Elton Plateau, 8500 feet, 6 Oct 1954, *R.L. Willan 172* (K). Near Njombe, 2100 m, 17 Jan 1957, *H.M. Richards 7874* (K). **Zambia:** Highlands, Ndola, Oct 1906, *C.F.E. Allen 363* (SRGH).

#### Specimen examined.

Tanzania, Nyassa Hochland, Station Kyimbila, Rmubya Braubuligebirge, 1300–1500 m Seehöhe, 25 Nov 1907, *A. Stolz 103* (US).

#### Habitat.

Evidently in savannah areas.

## Supplementary Material

XML Treatment for Lettowia

XML Treatment for Lettowia nyassae

## References

[B1] Anderson LE (1954) Hoyer’s solution as a rapid mounting medium for bryophytes. The Bryologist 57: 242-247

[B2] Chissoe WF Skvarla JJ (1996) Combining sputter coating with OTOTO treatment to eliminate charging artifacts in pollen preparations. Proceedings of the Oklahoma Academy of Science 76: 83-85

[B3] Chissoe WF Vezey EL Skvarla JJ (1995) The use of osmium-thiocarbohydrazide for structural stabilization and enhancement of secondary electron images in scanning electron microscopy of pollen. Grana 34: 317-324.10.1080/00173139509429065

[B4] Erdtman G (1960) The acetolysis method. A revised description. Svensk Botanisk Tidskrift 54: 561–564.

[B5] Gunther J (1956) Prussian Lion of Africa. True Magazine February issue: 33–35, 92–96.

[B6] Hoyt EP (1981) Guerilla; Colonel von Lettow-Vorbeck and Germany’s East African Empire. MacMillan, New York, London, i–iv, 1–216.

[B7] Jeffrey C (1988) The Vernonieae in East Tropical Africa. Kew Bulletin 43 (2): 195-277.10.2307/4113734

[B8] Lettow-Vorbeck PE von (1920) My reminiscences of East Africa. Hurst and Blackett, Paternoster House, London, 1–336.

[B9] Robinson H (1999a) Revisions in paleotropical Vernonieae (Asteraceae). Proceedings of the Biological Society of Washington 112 (1): 220-247

[B10] Robinson H (1999b) Generic and Subtribal Classification of American Vernonieae. Smithsonian Contributions to Botany 89: i–iv, 1–116.

[B11] Smith CE (1971) Observations on Stengelioid Species of *Vernonia*. Agricultural Handbook No. 396, U. S. Department of Agriculture, i–iv, 1–87.

